# No Blame No Gain? From a No Blame Culture to a Responsibility Culture in Medicine

**DOI:** 10.1111/japp.12433

**Published:** 2020-05-10

**Authors:** Joshua Parker, Ben Davies

**Affiliations:** ^1^ Wythenshawe Hospital Southmoor Road Wythenshawe Manchester M23 9LT UK; ^2^ Uehiro Centre for Practical Ethics University of Oxford Littlegate House, St Ebbe’s Street Oxford OX1 1PT UK

**Keywords:** Responsibility, blame, liability, professional ethics, medical ethics

## Abstract

Healthcare systems need to consider not only how to prevent error, but how to respond to errors when they occur. In the United Kingdom’s National Health Service, one strand of this latter response is the ‘No Blame Culture’, which draws attention from individuals and towards systems in the process of understanding an error. Defences of the No Blame Culture typically fail to distinguish between *blaming* someone and holding them responsible. This article argues for a ‘responsibility culture’, where healthcare professionals are held responsible in cases of foreseeable and avoidable errors. We demonstrate how healthcare professionals can justifiably be held responsible for their errors even though they work in challenging circumstances. We then review the idea of ‘responsibility without blame’, applying this to cases of error in healthcare. Sensitive to the undesirable effects of blaming healthcare professionals and to the moral significance of holding individuals accountable, we argue that a responsibility culture has significant advantages over a No Blame Culture due to its capacity to enhance patient safety and support medical professionals in learning from their mistakes, while also recognising and validating the legitimate sense of responsibility that many medical professionals feel following avoidable error, and motivating medical professionals to report errors.

## Introduction

Health care sometimes makes patients worse off. There is a robust body of evidence showing that errors and ‘adverse events’ within health care can harm patients. Such errors occur with worrying frequency: one systematic review claims that just under 10% of inpatient admissions are affected by an adverse event – ‘an unintended injury or complication…caused by healthcare management rather than by the patient’s underlying disease process’ – almost half of which are avoidable.^1^ Avoidable errors can cause minor harms, but they may also lead to significant harms; one report claims that 3.6% of deaths in acute hospitals are due to avoidable problems in care.^2^ This weight of error has seen the emergence of a ‘patient safety’ agenda in many countries, including the United Kingdom where successive governments since the 1990s have responded to scandals in the National Health Service (NHS) by implementing numerous policies to improve patient safety.^3^ The agenda emphasises both how to minimise error and how to respond to errors that occur.^4^ One strand of this latter response is developing ‘a culture that avoids a predisposition to blame’,^5^ or a ‘No Blame Culture’ (NBC). For instance, the UK government report *Learning Not Blaming* (2016) recommends five core principles.^6^ One of these, ‘Objectivity’, emphasises a focus on ‘learning and improvement’, rather than ‘find[ing] fault, attribut[ing] blame or hold[ing] people to account.’^7^ The NBC takes notions of blame off the table, drawing attention away from individuals in the process of understanding errors. Replacing this is a focus on systems. The goal is to redesign operations and processes to minimise error, accepting that human incompetence is rarely the sole cause of error and systems will typically amplify human fallibility.^8^

Defences of the No Blame Culture typically fail, however, to acknowledge an important distinction. This is the distinction between blaming someone and holding them responsible. As we argue in Section 2, while a person’s responsibility is *necessary* for permissibly blaming them, it is insufficient. In other words, one can be responsible, and yet not blameworthy. Our concern, therefore, is that a No Blame Culture risks throwing out the baby with the bathwater. In its focus on systems over individuals, it risks ignoring individuals’ responsibility within systems and minimising individual healthcare professionals’ own legitimate sense of responsibility when they make mistakes.

The case for a No Blame Culture relies on two main claims:

**‘Blame is Unjust’**: The system in which healthcare professionals work renders blame of individuals unfair, the system is to blame; and,

**‘Blame is Unsafe’**: Even if individuals are blameworthy, blame and the fear of blame is a bulwark to the openness and transparency that is required to improve patient safety.^9^

Each of these claims is sufficient but not necessary to justify a No Blame Culture. For instance, critics of the NBC note that a ‘systems approach’ cannot account for all errors. Healthcare professionals sometimes cut corners or disregard protocol.^10^ This presents a challenge to the claim that Blame is Unjust. But advocates of the NBC might insist that even if blame is just, we should not blame healthcare professionals because doing so will have bad consequences, i.e. Blame is Unsafe. Similarly, even if blaming people will not have the negative effects supposed by advocates of the NBC, it would still be wrong to blame healthcare professionals if it was true that doing so is unjust. A critique of the NBC must tackle *both* claims.

However, this raises the possibility that a culture that focuses on systemic issues may strike the wrong balance between the potential benefits of holding people accountable and the negative consequences of blame and fear of blame. While we agree that individual blame is often misplaced or excessive, we are sceptical that the No Blame Culture gets that balance right.

In this article, we argue for a ‘responsibility culture’ where healthcare professionals who make errors are held responsible in cases where errors meet both an epistemic condition of foreseeability and an agency condition of sufficient control. We use this account to demonstrate how healthcare professionals can justifiably be held responsible for their errors even if they work in challenging circumstances. In other words, we show that even if it is true in many cases that Blame is Unjust, this does not mean that it is unjust to hold someone responsible. We then review the idea of ‘responsibility without blame’, applying this to cases of error in healthcare. Sensitive to the undesirable effects of blaming healthcare professionals and to the moral significance of holding individuals accountable, we argue that a responsibility culture has significant advantages over a No Blame Culture in its capacity to both enhance patient safety and support medical professionals in learning from their mistakes, while also recognising the role that individuals play in error and the fact that this often demands that individuals take responsibility for their errors. Even if advocates of the NBC are right that Blame is Unsafe, it does not follow that holding people responsible is unsafe.

We are not the first to raise doubts about the NBC’s focus on systems over individuals. However, we offer a novel argument, first by considering not only the consequences of different focuses,^11^ but also their *fairness*. Second, our recommendation is broader than existing critiques, which tend to focus on *exceptions* to the NBC model, e.g. doctors whose *patterns* of behaviour may be identified as dangerous to patients.^12^ Finally, we theorise our alternative approach by reference to recent work in the philosophy of mental health, a facet that is absent from existing work.

We end this section by forestalling an obvious criticism. Since our arguments relate to claims about responsibility, advocates of the No Blame Culture might say that we are aiming at the wrong target: their arguments, after all, are about blame. Yet as we have observed, defences of the NBC *at least* fail to distinguish between the two concepts. What’s more, the typical suggestion that we focus on problems with systems – except in a small proportion of cases where healthcare professionals intentionally harm patients or repeatedly cut corners – suggests that many defences of the NBC conflate blame with responsibility. In either case, the link between our positive argument for a responsibility culture and criticism of the NBC seems clear.

## A Culture of Responsibility

The processes by which errors occur in health care are complex. When providing a single dose of a medication to an individual patient requires 80–200 separate steps,^13^ it is easy to see how understanding both simple and catastrophic errors is a mammoth task. Errors in health care have been divided into active and latent errors. Active errors are acts of individuals working directly with patients, including deviations from standards or protocols. Active errors can be made more likely by certain 14situations, e.g. if the individual is tired, stressed, or overwhelmed. With respect to a specific task, active errors include anything from forgetting a key part of that task to mistakes relating to the cognitive processes required to carry out that task.^15^ Latent errors, in contrast, are problems inherent in a system, often allowing or exacerbating active errors. For instance, problems in organisational structure may mean individuals are not aware that a task is their responsibility.

Within patient safety literature, it is recognised that active errors are an important cause of adverse events. However, many regard blaming individuals as unjustified, since it is rare that active errors occur in the absence of latent errors. Active errors are regarded as inevitable, not at the individual level (no particular active error is inevitable), but at the systemic level (given the existence of latent errors, it is inevitable that *some* active errors will occur).^16^ Despite the importance of latent errors, however, we suggest that active errors can imply a degree of responsibility.

Gerald Dworkin distinguishes between three types of responsibility. *Causal* responsibility occurs whenever an outcome can be causally traced to someone’s actions.^17^ However, you can cause an outcome and yet not hold *liability* responsibility: being answerable or accountable for your behaviour. For instance, it may be that your behaviour was unavoidable, or that you could not have predicted that it would cause the outcome. Finally, *role* responsibility concerns issues and areas that are under your auspices by virtue of a role you occupy. Robert Goodin refines this idea as ‘task responsibility’: ensuring that certain things do, or do not, happen because you have been assigned relevant tasks.^18^

We focus primarily on liability responsibility, and hence, ‘responsibility’ will refer to liability responsibility unless specified. The dominant framework for determining responsibility remains relatively unchanged since Aristotle, who outlines two conditions: agency and epistemic.^19^ The agency condition states that individuals cannot be responsible if there is sufficient outside interference or their action is in some way compelled. The epistemic condition stipulates that (nonculpable) ignorance of relevant information can also undermine responsibility. Insofar as somebody understands the consequences of their actions and has control over what they do, they are responsible. While people disagree on the specifics of each of these conditions, there is broad agreement that they are necessary in some form.

The two conditions come in degrees. Our beliefs about what we are doing, and what will result, vary in precision and degree of justification. Similarly, there are gradations of control; there is a difference between being *only just* in control, where any additional pressures will cause someone to lose control, and being firmly in control. There are also degrees of *difficulty* with which we exercise control. Having control over your behaviour can be mentally taxing or easy.^20^ Moreover, the two conditions can interact; an individual might fail to consider the ramifications of their actions *because* of external pressures placed on them. Perhaps due to finding themselves in an unfamiliar, stressful situation, a person could struggle to remember crucial information, whereas in other situations they could access it easily. Indeed, this seems to be the situation in which many healthcare professionals who make errors find themselves: their reasonable options are constrained by situational factors, while time pressures mean they cannot conceive of alternative possibilities.

We can now get a sense of what it would take to hold healthcare professionals responsible for their errors, while acknowledging the pressure they face. Where a healthcare professional is working in an environment that leaves them few options and little control over the outcome, their responsibility is diminished. If a healthcare professional could not know that their actions would harm a patient, then they should not be held responsible. However, where both epistemic opportunities and opportunities for control are more readily available, we have stronger grounds for holding people responsible. It is worth noting that the conditions described above are, strictly speaking, conditions for when a person *is* responsible. Our argument at this stage therefore offers a response to the claim that Blame is Unjust, namely that even if this is true, it does not follow that we should focus entirely on systemic flaws, because holding people *responsible* is not unjust.^21^

However, it is still possible that although medical professionals meet the criteria for liability responsibility, it would be inappropriate to *hold* them responsible (e.g. by communicating a judgement of responsibility, or by imposing professional sanctions) because it would do more harm than good. In this section and the next, we largely adopt a simplifying assumption, ignoring for the most part concerns about the harm done even by fairly holding someone responsible and assuming for the sake of argument that if there is good evidence that a person meets the criteria for liability responsibility, it is reasonable to hold them responsible. In other words, we will move at points during these sections from judgements that someone is responsible to considering appropriate ways of holding them responsible, without pausing to consider the broader challenge from the claim that Blame is Unsafe. We return to this broader challenge in the final section.

The picture sketched above suggests that both the epistemic and control conditions act as threshold concepts.^22^ Nonetheless, as we will suggest, that the two conditions come in degrees raises the possibility of gradated judgements: we need not judge as equivalent every individual who satisfies the epistemic and control conditions to some degree. Rather, we may need to regard differently those who make mistakes in circumstances that are difficult, but still controllable, and those who make mistakes that they could easily have avoided.

On our view, responsibility is therefore both a threshold concept and a concept that admits of degree. It is a threshold concept because there is a point below which a person cannot be said to be at all responsible, and this is not simply the point at which they *completely* lack control or relevant knowledge. But it is a degree concept because once a person has breached this threshold, we assume that our judgements about them and their decisions should still be sensitive to the degree to which they met the two conditions. Importantly, this is not simply determined by the situation itself, but also by a person’s individual position. This means that, for instance, a junior and senior doctor may face the same medical decision, and agree on the course of action, but that the senior doctor should be held more responsible^23^ because their experience gives them a greater epistemic standing, while their seniority gives them more control over the situation. We address this in further detail in the following section.

If both control and epistemic justification are threshold concepts, responsibility requires that healthcare professionals have a *sufficient degree* of justification and of control. Clearly, errors in health care are often complex, with multiple factors at play. Pointing to conditions that determine the exact point at which a healthcare professional becomes responsible in all cases will be impossible. Our point is that in many errors where healthcare professionals cause harm to a patient, the individual professional will have some contributory role to play. Contrary to the No Blame Culture, which considers the system as an entity devoid of actors with wills of their own, a responsibility culture considers individuals within the context of the system and does not deny their role in their errors. We can imagine an approach that considers both the individual and the system and interrogates the role each played, with responsibility being placed on healthcare professionals if they meet the epistemic and control conditions outlined.

So far, we have provided a framework for determining when healthcare professionals should be taken as responsible for an error by others. A distinct question is when healthcare professionals hold themselves responsible. Some healthcare professionals may be unfairly self‐critical, holding themselves responsible, and perhaps even blaming themselves, for decisions that were justified or excusable, e.g. because they could not reasonably have acted differently or because acting differently would have been extremely challenging.^24^ This may occur because the professional wonders whether there actually was something more they could have done, whether there was some factor they could have controlled, changing the outcome. Alternatively, a healthcare professional may recognise that nothing more could be done but nonetheless feel responsibility due to the nature of their relationship with the patient or their causal responsibility. This has parallels with Bernard Williams’ concept of ‘agent‐regret’:^25^ circumstances mean somebody lacked control over an outcome, but they nevertheless feel responsibility and the associated emotions.

It is not a requirement of a responsibility culture that people feel bad (in the sense of experiencing negative emotions) about the mistakes they have made. Nonetheless, a healthcare professional experiencing some level of negative emotion about a mistake may lead to good outcomes; for instance, victims of healthcare errors, and their families, may benefit from an apology from the individual healthcare professional involved, and this may be more likely to occur if the professional feels some sense of guilt or agent regret.

In addition, a responsibility culture acknowledges that no matter what our official policy, healthcare professionals often *will* hold themselves partly responsible for errors and that this will often include negative emotions. As we discuss in more detail below, a responsibility culture responds respectfully to healthcare professionals’ sincere moral reactions to errors in a way that the No Blame Culture fails to do.

However, we acknowledge that healthcare professionals can be too hard on themselves, holding themselves fully responsible for mistakes where they hold only partial responsibility. Part of the ideal of a responsibility culture is that where an error is partially explained, or exacerbated, by environmental working conditions, we extend understanding to the person who made the error. Encouraging them to take responsibility for their own part in a mistake must come with encouraging them to see which elements of the situation were *not* in their control.

## Being Responsible But Not Blameworthy

We have shown that healthcare professionals can reasonably be held responsible for some errors. Our central concern with the No Blame Culture is that its focus on systems obscures the important role of professional accountability. The risk is that in attempting to avoid the counterproductive results of blame, the NBC also loses sight of responsibility since, as we have suggested, the two are rarely distinguished and may be conflated.^26^ Here, we make the case that responsibility can be separated from notions of blame and stand alone.

That a person meets the conditions of liability responsibility is sometimes taken as a justification for blame. On this view, once we establish that somebody is responsible for a bad outcome, we then go on to blame them. In this way, blame and responsibility are related.

In our view, blame goes beyond holding someone responsible. To blame somebody requires not only that they are liable for an outcome, but a judgement that this reflects negatively on their desires, beliefs, motives, or character. Moreover, not just any negative judgement will do; blame is inappropriate in conditions where we recognise that although someone could and should have acted differently, they faced significant obstacles to doing so. This is because blaming someone involves a judgement of *significant* flaws in character, motivations, etc.

As we note in more detail below, some might adopt a far more minimal notion of blame, where being ‘blameworthy’ is equivalent to being responsible. Our view is that such a minimal notion of blame cannot ground the claim that Blame is Unjust. While it is often unjust to ascribe significant flaws in character, motivation, understanding, etc. to a medical professional who has made an error, it is not similarly unjust to hold them accountable for their role in an undesirable outcome, so long as we also acknowledge the obstacles they faced. This means that a minimal understanding of blame does not offer any support to the No Blame Culture.

Moreover, where liability responsibility will often bring with it certain requirements – e.g. to undergo further training, to reflect on one’s behaviour, to apologise otherwise to make amends – blame seems to be attached more readily to *punitive* burdens, i.e. burdens placed on an individual with the goal of punishment.^27^

We have sketched the conditions of responsibility and described what it is to blame somebody. When errors in health care occur, the current orthodoxy is that we ought not to blame healthcare professionals. The No Blame Culture therefore tends to focus on the system in its response to error, saving blame for the most egregious of errors. However, if we distinguish responsibility from blame, unlike the NBC, there is space to utilise responsibility appropriately and save pure systems analysis and notions of blame for those errors where healthcare professionals do not meet conditions for responsibility.

Of course, in highlighting the No Blame Culture’s overemphasis on systems, we do not reject the role of systems altogether. Even where an individual is not only responsible but blameworthy (e.g. if they deliberately harm a patient), there is a role for assessing the systems surrounding that behaviour, e.g. to assess whether different structures might have prevented the individual from acting as they did. Highlighting the role of individuals need not deny the role of systems, and acknowledging the role of systems need not deny the agency of the individuals who operate within them.

Our approach here is inspired by Hanna Pickard, who outlines how it is possible to hold individuals responsible without blaming them.^28^ Pickard applies this idea in the context of caring for individuals with addiction^29^ and personality disorder.^30^ However, she provides reasons to believe that blame and responsibility can be distinguished independently of attempting to help those with addiction or PD. Pickard charts a path between responsibility and blame by recognising that patients with PD and addiction have *diminished* rather than extinguished agency, making responsibility conceptually appropriate. What’s more, she also highlights the positive consequences of holding patients responsible. She makes her case by distinguishing where responsibility and blame originate. What Pickard calls *affective* blame concerns how we respond to someone who is responsible; it is about *our* emotions, judgments, and actions, whereas responsibility is about the person who has committed a wrong and what conditions must hold for them to take ownership of their behaviour.^31^ Pickard suggests that because affective blame is about our attitudes towards somebody else, we can decide whether we must also blame someone rather than merely holding them responsible. Pickard claims, ‘we can believe [somebody is responsible] *and hold people to account* – but not allow blame to infect our emotions, judgements, and actions towards them as a person. That is what it means to adopt the stance of Responsibility without Blame’.^32^ As Pickard’s experiences testify, it is possible to separate responsibility from blame both in theory and practice. Indeed, in her clinical practice, the attitude taken towards individuals with addiction or PD is to hold them responsible for wrong‐doing; this includes encouraging them to recognise that the behaviour was *their* behaviour, that they have the capacity to change, and that they are in some sense liable for the outcomes they cause. But Pickard also emphasises the importance of maintaining the typical emotions of a healthcare professional working with individuals who are unwell: compassion and understanding.

Pickard also provides reason to think that there are cases where an individual can be responsible without blame*worthiness*. There is a well‐subscribed view in philosophy that to be responsible is in some way reducible to being the appropriate *subject* of blaming and/or praising attitudes, even if these are not expressed.^33^ Pickard rejects this equivalence: in her view, one can be responsible but not blameworthy *when one has an excuse* (this means, as she notes, that responsibility does entail blameworthiness in the absence of excuses).^34^ We would add that the sense of blame that seems to operate in the claim that responsibility should be understood in terms of blameworthiness is a conceptually thin one, without any implied judgement of character or motive of the blameworthy individual. We have suggested that such a concept does not count as blame. But even if it does, it should be clear that it is not sufficient to ground the claim that Blame is Unjust. For while it may indeed be unjust to make negative judgements about the character, motives, or professionalism of healthcare workers who make errors in difficult circumstances, it is not clear why it should be unjust to make a more minimal judgement of responsibility.

The excuses that patients with personality disorders can offer clearly differ from the excuses available to medical professionals. But at the conceptual level, we believe there is much to be said for translating Pickard’s schema to a professional setting. The very obstacles that the No Blame Culture points to as a reason to discard responsibility altogether should, in our view, instead operate as excuses. Adopting a stance of responsibility without blameworthiness allows us to recognise and understand the complex causes of error in health care whilst viewing healthcare professionals as agents rather than mere passive participants of institutions and systems. Consider the following example:

Prescribing errors are very common and a cause of harm to patients.^35^ Dr. Jackson is working on a very busy and understaffed ward. It is his third day working on that ward, and he is finding it very stressful. Dr. Jackson prescribes a patient, Mary, penicillin, not realising that she has a penicillin allergy.

Dr. Jackson meets both the epistemic and control conditions and is therefore capable of exercising responsibility. There are many alternative antibiotics available to him; he knows (or should know) that some patients are allergic to penicillin, and this can have catastrophic consequences. While he lacks knowledge in one important respect – he does not know that prescribing Mary penicillin will lead to an allergic reaction – this is information that is easily available to him. Dr. Jackson should, and *could*, have done what most doctors would do and check whether the patient has an allergy before prescribing. For these reasons, he meets the conditions of responsibility.

On the other hand, we must also recognise that conditions were not ideal for Dr. Jackson to exercise agency. While working under pressure on a busy ward, in an unfamiliar environment and without support, does not make control impossible, it may well constrict it. The fact that Dr. Jackson was working under difficult circumstances means that we should withhold the attitudes and judgments associated with blaming. If Dr Jackson had faced no pressure, had plenty of time to think about his decision, or was under no doubt that this patient had an allergy, blame would be appropriate.

Dr. Jackson’s situation sits in the middle of these two. While he had alternative options, the pressure he was under meant that while he *should* have taken them, it is understandable why he failed to do so. As such, he should be encouraged to take responsibility for his error: to reflect on why it happened, his own role in that series of events, and how he can personally reduce the likelihood of it happening again. Nonetheless, others should not take this mistake to reflect poorly on Dr. Jackson’s *character*, *motives,* or even *general professional capability.* Dr. Jackson does not deserve negative character appraisal or negative emotions (though it is understandable if Mary and her family do not see things this way). Nor does he deserve formal punishment. This is the essence of applying responsibility without blame in a healthcare context. Many errors in healthcare are of a kind with Dr Jackson’s case: the individual healthcare professional retains both agency and the opportunity for relevant knowledge and hence can be held responsible, while an appreciation of the conditions under which errors occurred makes blame inappropriate.

Pickard also argues that an attitude of compassion should prevail when applying responsibility without blame. This too can be extended to errors in health care. In a sense, adopting a stance of compassion acknowledges that the No Blame Culture gets something right: responding to errors in an overcritical way is both unfair and risks negative consequences by demoralising healthcare professionals.

What does it mean to show compassion?^36^ While it may be inappropriate to talk strictly of institutions as expressing compassion – unable as they are to feel sympathy – we may talk, somewhat metaphorically, of encouraging a ‘culture of compassion’ within an institution such as a healthcare system. As we understand it, compassion at an institutional level involves at least three things. First, to show compassion towards someone involves demonstrating understanding. In the case of errors by healthcare professionals, this may involve understanding that the person who has made a mistake may now be in significant distress, and suffering because of their error.^37^^,^ 38 However, it may involve an understanding of the difficulties the healthcare professional faced in making their decision.

Second, a compassionate response requires that this understanding leads to sympathy/empathy for their situation. It is not enough to understand what a person is going through at a theoretical or intellectual level. A compassionate response also requires that the relevant features (i.e. the suffering and/or constraints on judgement) are considered in further decision‐making. This is where we can talk (metaphorically) about an institution such as the NHS ‘showing compassion’, or ‘having a culture of compassion’. Even if an institution cannot literally feel compassion, policies and procedures can be designed in a compassionate way, and this will include reflecting the appropriate features of a healthcare professional’s error in the institutional response to that error, as well as encouraging colleagues and superiors to show interpersonal compassion.

Finally, in our view compassion requires that the subject of compassion is given appropriate *support*. What form this support should take will depend on the particular aspects of the healthcare professional, and the error for which they are responsible. It may be that they require support in coming to terms with their own role in the harming of a patient. Alternatively, support may require a more formal institutional response, such as the provision of additional or remedial training. Finally, support can come in helping a healthcare professional to begin the process of understanding and addressing their own responsibility for the error. For instance, in our scenario above, Dr Jackson might feel upset and embarrassed by his mistake and its effect on the patient. These feelings could be managed, and further support in coping should be offered. In addition, Dr Jackson could be helped to understand *both* how various systemic factors contributed to error *and* how his own decisions and behaviour within those systems made error more likely. An approach that focuses solely on systems cannot help medical professionals properly come to terms with their individual role in error. His role in the error occurring might mean he must demonstrate his understanding of antibiotics and allergy and accept training if this can be improved.

It is important to note the consistency between our claim that a responsibility culture will often avoid *punishing* a medical professional and the idea that those who make errors might be required to take certain actions, such as explicitly reflecting on what went wrong or undertaking additional training. To punish someone involves, in our view, an expression of condemnation; punishments are penalties that are applied *because someone has done wrong*. As such, an absence of punishment does not mean that errors are consequence free. But what is important is to communicate the reasons behind the required action.

We acknowledge the distinction between being responsible for an error and being to blame for it is at risk of getting lost in the day‐to‐day practice of health care. Since what we are advocating is a change in *culture*, it is therefore important that this distinction is reinforced both during training on liability that medical professionals already undergo and in explicit communication to those who have made errors. For instance, one part of the institutional response to an error might be to explain to the relevant professional that, ‘We want to emphasise that you are not being blamed for this; however, we want to encourage and help you to take responsibility for what you could have done differently’. We cannot pretend that things will never go wrong – so much is true of any institutional culture – but we do believe that much can be done to minimise this eventuality.

## Responsibility and Blame in Healthcare Error

Healthcare professionals can be held responsible for many of their errors without this developing into blame. However, as we noted in Section 1, defenders of a No Blame Culture might accept that healthcare professionals can be responsible for errors (against the claim that Blame is Unjust), and yet in practice reject holding healthcare professionals responsible because doing so will do more harm than good (Blame is Unsafe). If we are going to suggest that healthcare professionals should be held responsible, it is therefore not enough to show that they *are* responsible; we also need to demonstrate that holding them responsible will, on balance, have good effects. We have already made some progress towards this goal, e.g. in our suggestion that accepting the possibility of individual responsibility may valuably acknowledge a legitimate sense of responsibility on the part of a medical professional who makes an error and legitimate individual grievances on the part of patients and their loved ones. This section outlines a further relevant issue, namely how each system motivates and supports the reporting of error. Since this is the most significant evidence used to support the claim that Blame is Unsafe, meeting this challenge provides us with a good basis on which to defend the Responsibility Culture.

The concern with data collection in order to understand error shown by supporters^39^ of the NBC is laudable, and it is understandable how concerns about the negative impact of blame for healthcare professionals could hinder disclosure of ‘near misses’ and adverse events. While the reasons that healthcare professionals fail to report go beyond blame,^40^ reducing blame is a reasonable step.

However, it is important to note that removing blame does not by itself *provide* a motivation to report mistakes. As we have already discussed, it is quite natural when involved in a mistake to respond by feeling culpability, guilt, and other negative emotions. To the extent that healthcare professionals feel ashamed of their part in an error, there may still be some reluctance to report. Moreover, an environment that focuses on systems offers no place for these feelings, except that they are mistaken. In other words, a No Blame Culture denies healthcare professionals their own agency and invalidates their feelings of responsibility. It also risks failing to provide positive motivation to report, instead relying on healthcare professionals’ sense of obligation. A responsibility culture, on the other hand, motivates reporting because it encourages medical professionals to take responsibility for their mistakes. Part of what it means to be responsible for a mistake is that one has an obligation to take an active role in either undoing it or making amends.

Defenders of the No Blame Culture might respond in two ways. First, they might claim that even if there is no positive motivation to report errors inherent in the NBC, it is not inconsistent with it. On this view, although we refuse to blame healthcare professionals for their mistakes, we should hold them to an obligation to *report* mistakes. But this response faces a regress: how should we respond to someone who fails to report an error? After all, this failure might itself be ascribable to systemic problems. Defenders of the NBC must either say that we should hold such an individual responsible, or not. If we do not hold them responsible, then the No Blame Culture still faces a motivational gap. If we *do* hold them responsible, it is unclear why we cannot also hold people responsible for at least some first‐order errors.

The second possible response is an empirical one. Defenders of a No Blame Culture in medicine sometimes point to analogous successes in other sectors, such as aviation.^41^ Since the NBC in aviation has not led to significant failures to report, why expect this in medicine?

We agree that the success of a No Blame Culture in aviation provides some support for its implementation in other areas. However, we are sceptical about this analogy. As we have said, it is common and understandable for medical professionals to feel bad when they make mistakes, particularly when a patient is harmed, or could have been harmed. This seems natural: although the precise details vary, medical professionals have a direct *relationship* with their patients, in a way that aviation workers do not have with the individuals who would be affected by error. What’s more, the negative feelings that seem natural in cases of medical error – guilt, self‐recrimination, shame – affect people’s motivations in different ways. Guilt can make us want to admit our mistakes in the hope of forgiveness. But guilt and shame can make us want to hide responsibility, particularly where we feel that forgiveness is unlikely. Although we admit that this is speculative, and not backed up by empirical work, this seems to us an important difference between the two sectors that should at least make us think again about the support provided by an analogy with aviation.

The No Blame Culture does not encourage healthcare professionals to feel these emotions. But in its focus on the role of systems, it also fails to take these emotions seriously. In contrast, a responsibility culture takes seriously the fact that healthcare professionals may feel badly about their errors and acknowledges that those feelings are significant and may be appropriate. Since it also offers support to individuals who have made mistakes, it encourages healthcare professionals to *take* responsibility for their errors, which starts with reporting them.

It can also avoid the errors of a ‘blame culture’, where fear of blame and punishment may provide an alternative reason not to report error. Given that one can be held responsible for one’s actions even if no bad outcome occurs, a responsibility culture allows us to capture ‘near misses’ as well as errors that actually harm patients. Since compassion is also part of the institutional response to error, a responsibility culture offers extra reassurances to healthcare professionals not only that they won’t be treated negatively and blamed, but that in engaging with their institution they should find compassionate understanding, but also a recognition that their errors have implications for them.

Errors in health care impact patients. Whilst it is important to prevent patients coming to harm, the response to error cannot *only* be learning. Where a patient has been harmed by error they are owed an apology, but there is also risk of damage to the professional‐patient relationship and to trust in the profession. While it is true that the process of learning and preventing further errors could bolster trust in the profession, this does not negate the need for an apology. As others have suggested, this could entail ‘prospective responsibility’ by disclosing error, offering apologies and considering future improvements.^42^ For instance, Daniel Tigard has argued that ‘taking the blame’ is important in the disclosure of error in health care to facilitate these kinds of reparations.^43^ He argues that self‐blame motivates certain moral emotions, including regret, remorse, and guilt, which are required to demonstrate that a healthcare professional is committed to personal and institutional improvements in addition to repairing their relationship with their patients and fostering trust. In our view, Tigard’s argument applies more readily to taking responsibility than to ‘taking the blame’. To repeat the point made in Section 2, while we agree that negative emotions can have an important motivating effect, they can also have the effect of discouraging people from reporting and also can adversely affect mental health and wellbeing.

## Conclusion

In this article, we have argued for a responsibility culture that embraces the complexity of medical error, but which acknowledges the role that healthcare professionals play in it. While we have not ruled out blaming healthcare professionals in some cases, we have sought to find a middle path through routinely blaming healthcare professionals on the one hand, and the No Blame Culture on the other. In doing so, we have argued that blame can be distinguished from responsibility and that in doing so distinct advantages both of the NBC and those omitted by this can be attained.

Error in health care is a significant and complex cause of patient morbidity and mortality. It’s important that we get the response to this right, both as individuals and at the institutional level. Our responses matter both in terms of dealing with error after the fact and preventing future error. We believe that there is much that the No Blame Culture gets right in terms of acknowledging the role of systems in errors and a commitment to preventing future error. The problem for the NBC is that it fails to account for the role of individuals in healthcare error and thereby misses an opportunity to capture the benefits of taking an individualised stance on error, such as making reparations, fostering personal growth, and taking seriously medical professionals’ own feelings of responsibility. By distinguishing blame from responsibility and advocating for a responsibility culture, we are better able to accommodate the full range of responses to error in health care in terms of repairing the relationship to the patient, developing as an individual, and taking steps towards preventing future error.

## Notes

1 E.N. de Vries, M. A. Ramrattan, S. M. Smorenburg, D. J. Gouma, and M. A. Boermeester, ‘The incidence and nature of in‐hospital adverse events: A systematic review’, *BMJ Quality & Safety* 17 (2008): 216–23, p.216.

2 Helen Hogan, Rebecca Zipfel, Jenny Neuburger, Andrew Hutchings, Ara Darzi, and Nick Black, ‘Avoidability of hospital deaths and association with hospital‐wide mortality ratios: retrospective case record review and regression analysis’. *BMJ* 351, h3239 (2015).

3 Chris Ham, Don Berwick and Jennifer Dixon, *Improving Quality in the English NHS: A Strategy for Action* (London: The King’s Fund, 2016). URL=https://www.kingsfund.org.uk/sites/default/files/field/field_publication_file/Improving‐quality‐Kings‐Fund‐February‐2016.pdf. Accessed 27 March 2019.

4 Linda Emanuel, Don Berwick, James Conway, John Combes, Martin Hatlie, Lucian Leape, James Reason, Paul Schyve, Charles Vincent, and Merrilyn Walton, ‘What exactly is patient safety?’ in Kerm Henriksen, James B. Battles, Margaret A. Keyes, and Mary L. Grady (eds.) *Advances in Patient Safety: New Directions and Alternative Approaches (Vol. 1: Assessment)* (Rockville (MD): Agency for Healthcare Research and Quality, 2008).

5 National Advisory Group on the Safety of Patients in England, *A promise to learn – a commitment to act.* Crown copyright 2901213. 2013: p. 9. https://assets.publishing.service.gov.uk/government/uploads/system/uploads/attachment_data/file/226703/Berwick_Report.pdf. Accessed 25 April 2019

6 While this article focuses on the UK context through the framework of the National Health Service, our arguments apply to the idea of a No Blame Culture wherever it is implemented.

7 Department of Health, *Learning Not Blaming: The Government Response to the Freedom to Speak Up Consultation, the Public Administration Select Committee Report ‘Investigating Clinical Incidents in the NHS’ and the Morecambe Bay Investigation,* 2015. URL=https://assets.publishing.service.gov.uk/government/uploads/system/uploads/attachment_data/file/445640/Learning_not_blaming_acc.pdf. Accessed 13 March 2019.

8 M. Walton, ‘Creating a “no blame” culture: Have we got the balance right?’, *BMJ Quality & Safety* 13, 3 (2004): 163–4.

9 James Reason, ‘Human error: Models and management’ *The BMJ* 320, 768 (2000).

10 Walton, op. cit.

11 Donald Goldmann, ‘System failure versus personal accountability — The case for clean hands’, *The New England Journal of Medicine*, 355 (2006): 121–123

12 Lucian Leape and John Fromson, ‘Problem doctors: Is there a system‐level solution?’ *Annals of Internal Medicine*, 144, 2 (2006): 107–115; Robert Wachter and Peter Pronovost, ‘Balancing “no blame” with accountability in patient safety’, *The New England Journal of Medicine* 361 (2009): 1401–1406

13 No Author, ‘Pharmacy‐nursing shared vision for safe medication use in hospitals: Executive summary session’, *American Journal of Health‐System Pharmacy* 60, 10 (2003): 1046–52.

14 James Reason, ‘Safety in the Operating Theatre – Part 2: Human Error and Organisational Failure*’. *Quality & Safety in Health Care* 14, 1 (2005): 56–60.

15 Eric Moyen, Eric Camiré, and Henry Thomas Stelfox, ‘Clinical review: Medication errors in critical care’, *Critical Care* 12, 2 (2008): 208.

16 Reason, op. cit.

17 Gerald Dworkin, ‘Taking risks, assessing responsibility’. *The Hastings Center Report* 11, 5 (1981): 26–31.

18 Robert E. Goodin, ‘Apportioning responsibilities’. *Law and Philosophy* 6, 2 (1987): 167–85.

19 Andrew Eshleman, ‘Moral responsibility’ in Edward N. Zalta (ed.) *The Stanford Encyclopedia of Philosophy*. (2016) URL = https://plato.stanford.edu/archives/win2016/entries/moral‐responsibility, Accessed 15 March 2019

20 D. Justin Coates and Philip Swenson, ‘Reasons‐responsiveness and degrees of responsibility’, *Philosophical Studies* 165, 2 (2013): 625–49, p. 634; Dana Nelkin, ‘Difficulty and degrees of moral praiseworthiness and blameworthiness’, *Noûs* 50, 2 (2016): 358–78.

21 We have, of course, relied on a particular conception of the conditions of responsibility. These conditions are contestable, although they are widely endorsed. However, our broader point is that it does not follow from the claim that Blame is Unjust that we cannot hold medical professionals responsible.

22 Fischer and Ravizza, op. cit.

23 Some authors (e.g. Fischer and Ravizza, op. cit.) deny that responsibility can come in degrees, although they acknowledge that related concepts such as blameworthiness can do so. Since we are explicitly *not* discussing blame at this point, but still want to maintain that degrees of control and epistemic access can make a difference, we are inclined to disagree. However, we note that even if one wants to hold responsibility as a purely threshold concept, one might instead interpret our argument as supporting gradations in *nonblame* responses, i.e. in *holding* someone responsible.

24 Whether the same standards apply to holding someone else responsible, and holding oneself responsible, is unclear. It at least seems possible that there can be situations where it is apt for a healthcare professional to hold themselves responsible, but where it would not be appropriate for, e.g. colleagues or superiors to *communicate* a judgement of responsibility (one possible meaning of ‘holding responsible’), either formally or informally.

25 Bernard Williams, *Moral Luck* Cambridge: Cambridge University Press, p.29

26 In a 2016 speech, then‐Health Secretary Jeremy Hunt noted that ‘relatives who had suffered [from serious mistakes] cry out in frustration that no one had been “held accountable”’. However, he added, ‘to blame failures in care on doctors and nurses trying to do their best is to miss the point that bad mistakes can be made by good people. What is often overlooked is proper study of the environment and systems in which mistakes happen and to understand what went wrong and encouragement to spread any lessons learned’. See Department of Health and The Rt Hon Jeremy Hunt MP, *From a blame culture to a learning culture,* 10 March 2016. URL = https://www.gov.uk/government/speeches/from‐a‐blame‐culture‐to‐a‐learning‐culture. Accessed 15 March 2019. Similarly, the previously mentioned *Learning, Not Blaming* report (Department of Health, op. cit.) champions a focus on learning from mistakes, rather than blaming *or* ‘hold[ing] people to account’. These are both conflations of blame and responsibility. Other defences of the No Blame Culture do not so explicitly conflate the two ideas, but they fail to acknowledge the distinction.

27 Neal Tognazzini and D. Justin Coates, ‘Blame’ in Edward N. Zalta (ed.) *The Stanford Encyclopedia of Philosophy*, 2018. URL= https://plato.stanford.edu/archives/fall2018/entries/blame/ Accessed 25 April 2019.

28 Hanna Pickard, ‘Responsibility without blame: Therapy, philosophy, law’, *Prison Service Journal* 213 (2014): 10–16.

29 Hanna Pickard, ‘Responsibility without blame for addiction’, *Neuroethics* 10, 1 (2017): 169–80.

30 Hanna Pickard, ‘Responsibility without blame: Empathy and the effective treatment of personality disorder’, *Philosophy, Psychiatry, & Psychology* 18, 3 (2011): 209–3.

31 Pickard, op. cit 2014.

32 ibid. p. 14.

33 John Martin Fischer and Mark Ravizza, *Responsibility and Control: A Theory of Moral Responsibility*. (Cambridge: Cambridge University Press, 1998); Shoemaker, D. (2015). Responsibility from the Margins. Oxford: Oxford University Press; Gary Watson (1993). ‘Responsibility and the limits of evil: Variations on a strawsonian theme,’ in J. M. Fischer and M. Ravizza (eds) Perspectives on Moral Responsibility. Ithaca, NY: Cornell University Press, pp. 119–148.

34 Hanna Pickard, ‘Responsibility without blame: philosophical reflections on clinical practice’ KWM Fulford *et al*., eds. *Oxford Handbook of Philosophy and Psychiatry:* 1134–1152 (2013), p. 1142

35 B. Dean, M. Schachter, C. Vincent, and N. Barber, ‘Prescribing errors in hospital inpatients: Their incidence and clinical significance’. *Quality & Safety in Health Care* 11, 4 (2002): 340–4.

36 One might wonder why, having endorsed Pickard’s general line of thinking, we do not simply endorse her view of compassion. Part of the answer is that Pickard does not offer an explicit analysis of compassion and seems to rely instead on a common‐sense view of the term. In addition, Pickard is explicitly focused on interpersonal relationships; we also want an account of compassion that applies (even if only metaphorically) to institutions, e.g. through the claim that institutions and policies should show compassion to healthcare professionals when they make mistakes.

37 Martha Nussbaum endorses a recognition of suffering as an essential judgement in compassion. We disagree – someone may not suffer from having made an error, since they recognise the difficulty they were under. See Martha Nussbaum, *Upheavals of Thought: The Intelligence of Emotions.* (Cambridge: Cambridge University Press, 2001), p.304

38 Some have even described healthcare professionals as the ‘second victim’ of an error. E.g., Albert Wu ‘Medical error: The second victim’, *BMJ* 320, 7237 (2000): 726–7. This idea has come under considerable criticism, e.g. Melissa Clarkson, Helen Haskell, Carole Hemmelgarn, and Patty Skolnik, ‘Abandon the term “second victim”’, *BMJ* 364 (2019): 1233.

39 Paul Barach and Stephen D. Small, ‘How the NHS can improve safety and learning. By learning free lessons from near misses’, *BMJ* 320, 7251 (2000): 1683–1684; J. Firth‐Cozens, ‘Barriers to incident reporting’ *Quality & Safety in Health Care*, 11, 1 (2002): 7.

40 Justin J. Waring, ‘Beyond blame: Cultural barriers to medical incident reporting’, *Social Science & Medicine* 60, 9 (2005): 1927–35.

41 e.g. Reason, op cit.

42 Nancy Berlinger and Albert Wu, ‘Subtracting insult from injury: Addressing cultural expectations in the disclosure of medical error’, *Journal of Medical Ethics* 31, 2 (2005): 106–8.

43 Daniel W. Tigard, ‘Taking the blame: Appropriate responses to medical error’. *Journal of Medical Ethics* 45, 2 (2019): 101–5.

